# Overdriven laser diode optoacoustic microscopy

**DOI:** 10.1038/s41598-023-46855-w

**Published:** 2023-11-09

**Authors:** Markus Seeger, Antonios Stylogiannis, Ludwig Prade, Sarah Glasl, Vasilis Ntziachristos

**Affiliations:** 1 Chair of Biological Imaging at the Central Institute for Translational Cancer Research (TranslaTUM), School of Medicine, Technical University of Munich, Ismaninger St 22, 81675 Munich, Germany; 2 Institute of Biological and Medical Imaging, Helmholtz Zentrum München, Ingolstädter Landst. 1, 85764 Neuherberg, Germany; 3 Munich Institute of Biomedical Engineering (MIBE), Technical University of Munich, Boltzmannstr. 11, 85748 Garching b. München, Germany

**Keywords:** Biological techniques, Optics and photonics

## Abstract

Laser diodes are small and inexpensive but don’t afford the pulse energy and beam profile required for optoacoustic (photoacoustic) microscopy. Using two novel modulation concepts, i.e. overdriving continuous-wave laser diodes (CWLD) and frequency-wavelength multiplexing (FWM) based on illumination pulse-trains, we demonstrate concurrent multi-wavelength optoacoustic microscopy with signal-to-noise ratios of > 17 dB, < 2 µm resolution at repetition rates of 1 MHz. This unprecedented performance based on an adaptable trigger engine allowed us to contrast FWM to wavelength alternating acquisition using identical optical components. We showcase this concept’s superiority over conventional optoacoustic microscopes by visualizing vascular oxygenation dynamics and circulating tumor cells in mice. This work positions laser diodes as a technology allowing affordable, tunable, and miniaturizable optoacoustic microscopy.

## Introduction

Optical-resolution optoacoustic (photoacoustic) microscopy (OR-OAM) is uniquely suited for the dynamic and label-free study of biological processes at high resolution and speed by accessing intrinsic contrast of multiple biological moieties. Time-domain OR-OAM typically operates using nano-second light pulses with pulse energy in the ~ 10–100 nJ range and repetition rates of up to 1 MHz^1–3^. Such excitation specifications are usually provided by diode-pumped solid-state (DPSS) lasers in combination with optical-parametric-oscillators (OPOs) or dye-units for wavelength tuning^3–6^. However, DPSS lasers with OPOs or dye-units are slow, complex, costly, generally bulky, and can exhibit marked pulse-to-pulse fluctuations. Importantly, since they can only illuminate at one wavelength at a time, imaging at multiple wavelengths requires sequential acquisition of images at each desired wavelength, after tuning the laser to each wavelength of interest. The tuning requirement increases the overall imaging time, may limit the image fidelity due to sample movement, and prevents monitoring of fast processes. Integrating multiple discrete laser sources for each excitation wavelength, to overcome the necessity for slow wavelength tuning, increases the cost and size of the optical assembly. Therefore, traditional DPSS laser sources impede both miniaturization and the design of a cost-effective implementation of simultaneous multi-wavelength optoacoustic microscopy.

The utility and versatility of optoacoustic microscopy could markedly improve with illumination based on inexpensive sources as it would allow the development of affordable, portable, and application-tailored imaging devices in pre-clinical and clinical settings^7,8^. Lower cost and flexible optoacoustic microscopy could enhance diagnosis, treatment monitoring, and drug development by giving more researchers and physicians access to label-free biomolecular contrast at cellular resolution.

Pulsed laser diodes (PLDs) and continuous-wave laser diodes (CWLDs), have been considered as inexpensive alternatives for illumination in optoacoustic microscopy. Laser diodes have been used to provide optical excitation for optoacoustic signal generation with a small footprint, low cost, and without requiring extensive cooling while providing stable operation^7,8^. PLDs have demonstrated OR-OAM imaging with pulse repetitions up to 20 kHz^9^, pulse lengths down to 55 ns^9^, pulse energies of 50 nJ^8,10^ and with signal-to-noise ratios (SNRs) of 22 dB^10,11^. Despite a relatively large emitting area of up to ~ 400 × 800 µm^2^^7,11^, PLDs have achieved lateral resolutions of ~ 1.0 µm via diffraction-limited focusing^10^ in OR-OAM, and have been applied for imaging phantoms and ex vivo samples. Nevertheless, PLDs are typically available only in the near-infrared range^7,12^, which limits their applicability due to the low absorption of intrinsic chromophores in that spectral range. CWLDs achieve pulse repetitions up to 40 kHz^13^ or sinusoidal modulation up to 6 MHz^14^, pulse lengths down to ~ 7 ns^15^, and pulse energies of up to 52 nJ^10^. CWLDs have a broader wavelength availability across the ultra-violet and near-infrared spectral range^7,15,16^ and are associated with small emitting areas of up to approximately 1 × 100 µm^2^. CWLDs have achieved lateral resolutions of 0.95 µm via diffraction-limited focusing in OR-OAM and have been applied to in vivo imaging applications as well in mice^17^. However, the low pulse energies combined with a low repetition rate have so far necessitated significant averaging of up to 3000 times^17^ to yield OR-OAM imaging SNRs of 20 dB, which leads to long acquisition times^10,12,15–18^. PLDs and modulated CWLDs represent an interesting excitation alternative for OR-OAM, especially since multiple laser diodes can be implemented for concurrent multi-wavelength imaging in a cost-effective manner compared to solid-state laser sources. Nevertheless, there has been limited use of laser diodes for OR-OAM applications, due to the low energy outputs offered by currently available LDs, compared to other laser sources^7,8^. Moreover, LD-based OR-OAM has been so far only implemented at single excitation wavelengths, mainly in the 700–1000 nm region^9,14,19–21^ or at 405 nm^10,17^.

Herein we aimed to develop a laser-diode based optoacoustic microscopy for high performance applications, well beyond the current state of the art. To optimize the characteristics over any previous implementation, we employed two novel modulation schemes as follows. First, we overdrove CWLDs to provide optical pulses of high energy (up to 200 nJ/pulse), low duration (down to 10 ns/pulse), and Gaussian-shaped beam profiles, an approach that we have recently introduced which demonstrated efficient photon generation without longevity loss^16,22,23^, surpassing previous attempts using laser diodes for optoacoustic imaging. Second, we employed the novel concept of Frequency-Wavelength Multiplexing (FWM), an approach that uses pulse trains to generate multiple-discrete frequencies, in analogy to frequency comb methods but in the time–frequency domain^32^. FWM allows simultaneous illumination with multiple wavelengths, thus avoiding time-sharing and improving the SNR achieved as a function of the square root of the number of wavelengths employed. FWM is ideally suited for use with laser diodes, due to the CWLD availability at abundant wavelengths across the visible and near-infrared spectrum. In other words, FWM not only enables multi-wavelength illumination for spectral measurements but it does so with simultaneous illumination of all lines, avoiding slow tuning processes as common in DPSS lasers with OPOs or dye-units.

This never-before-achieved implementation of overdriven CWLDs was demonstrated herein with developing a novel microscope comprising three CWLDs, each equipped with a custom-built driver and control circuit. Precise optical assembly enabled < 2 µm near-diffraction-limited resolution and > 17 dB SNR at speeds of up to 1 MHz repetition rate. Our laser diode-based excitation achieves, for the first time, truly-simultaneous multi-wavelength OR-OAM at high frame rates using miniature and cost-effective diodes with imaging performances similar to or beyond traditional laser sources. We capitalized on this new performance and monitored, using low-cost CWLDs for the first time, oxygenation stress tests and circulating tumor cells label-free in live mice, at multiple wavelengths and high spatial and temporal precision. Additionally, while existing OR-OAM systems are optimized for one acquisition mode, the adaptable triggering engine (ATE) of the CWLDs allowed us to investigate two representative acquisition modes for simultaneous multi-wavelength imaging using the identical components: alternating laser excitation (ALEX) and frequency wavelength multiplexed (FWM) signal detection^32^. Our data demonstrate the true potential of semiconductor light sources as an economically and logistically improved alternative to other laser sources while also paving the way for low-cost and miniaturizable multi-wavelength optoacoustic imaging for microscopy applications.

## Results

**Figure 1:**
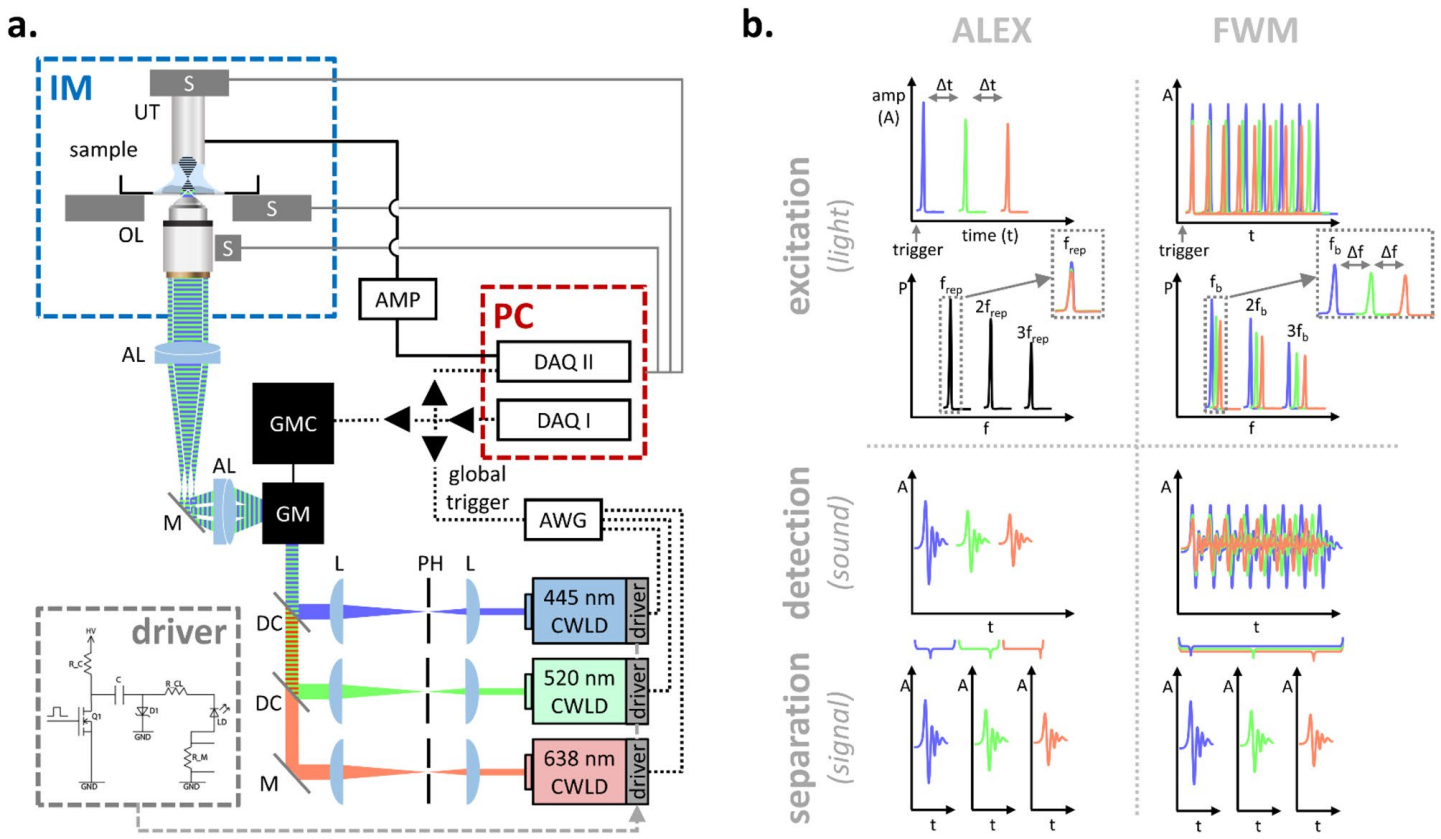
Schematic depiction of the CWLD system for high-speed ALEX and FWM optoacoustic microscopy. (**a**) The system equips three CWLDs, each of which was controlled by a custom-designed electrical circuit driver and received ultrashort trigger signals provided by arbitrary function generators. AL, achromatic doublet lens; AMP, low noise amplifier; AWG, arbitrary waveform function generator; CWLD, continuous wave laser diode; DAQ, data acquisition card; GM, galvanometric mirror scanner; GMC, GM controller; IM, inverted microscope; L, plano-convex lens; M, dielectric mirror; OL, microscope objective lens; PH, pinhole; S, high-precision motorized stage; UT, ultrasound transducer. (**b**) Principle of using the CWLD for alternating laser excitation (ALEX) or Frequency-Wavelength Multiplexed (FWM) optoacoustic microscopy. (Driver inset adapted from^16^).

The integration of three CWLDs for optoacoustic microscopy operating in ALEX- or FWM-mode is schematically depicted in Fig. 1.

Our system consists of three integrated CWLDs, each with identical electrical as well as optical settings (Fig. 1a). The CWLDs were driven by a custom-designed electrical circuit (see inset in Fig. 1a) as comprehensively described previously^16^. This driver could achieve current pulses shorter than ~ 8 ns of up to ~ 50–60 A at repetition rates surpassing ~ 500 kHz. The trigger-TTL-signals for the CWLDs were provided by two electrically synchronized high-speed arbitrary waveform function generators (AWG) serving as an adaptable-triggering engine (ATE), which allowed for multi-wavelength optoacoustic microscopy based on two approaches—ALEX and FWM—as schematically illustrated in Fig. 1b. ALEX led to firing all diodes sequentially one after another upon each global trigger signal. The frequency-power-spectrum of the optical excitation contained the underlying trigger signal repetition rate *f*_*rep*_ as well as higher harmonic occurrences. Due to *f*_*rep*_ ≈ 100–200 kHz, the data were acquired exclusively at *f*_*rep*_ and contained time-shifted OA signals corresponding to the excitation of the three CWLDs. Based on the applied *Δt* in their time-point of emission, the OA signals could then be separated from each other by time-windowing. FWM drove the CWLDs simultaneously in pulse trains (i.e., bursts) at different burst repetition rates of *f*_*b*_ *+ n·Δf*. Therefore, the frequency-power-spectrum contained three sets of frequency harmonics, in which their respective peak locations deviate at increasing frequency harmonics.

**Figure 2:**
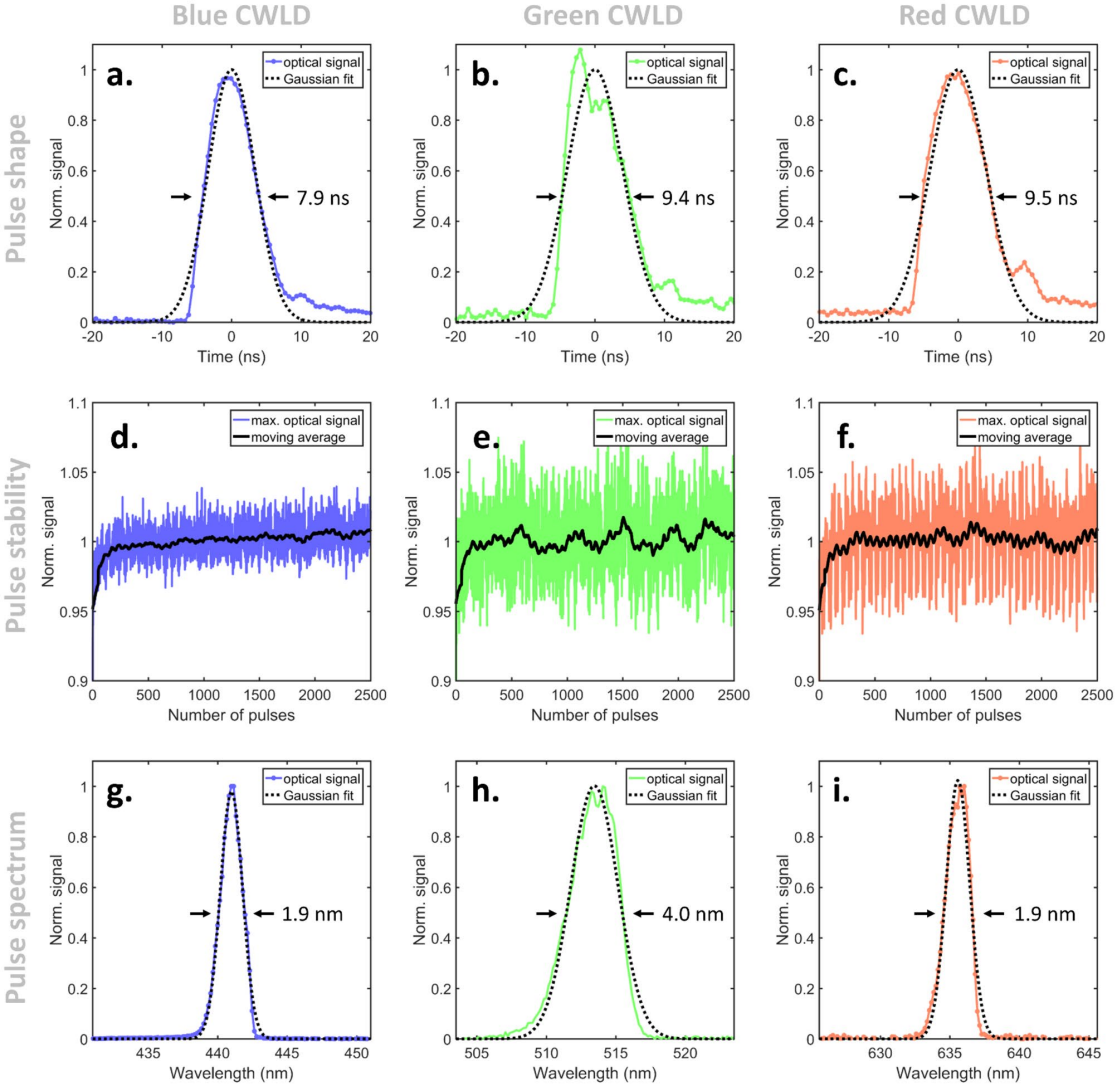
Optical characterization of overdriven CWLDs for ALEX and FWM optoacoustic microscopy. Performance occurred at 100 kHz repetition rate, 16 ns electric trigger signals, and 300 V high-voltage supply. (**a**–**c**) Optical pulse shapes of the (**a**) blue, (**b**) green, and (**c**) red CWLD. (**d**–**f**) Pulse energy stability of the (**d**) blue, (**e**) green, and (**f**) red CWLD. (**g**–**i**) Emitted wavelength of the (**g**) blue, (**h**) green, and (**i**) red CWLD.

Since the attempt to overdrive CWLDs in a pulsed mode is beyond their intended method of operation, we first characterized the performance of the CWLDs for both the ALEX and the FWM approach. As each CWLD driver powers the diodes with ultra-short electrical pulses, electrical cross-talk among the diodes could not be excluded. For that, all CWLDs were operating simultaneously during the characterization according to the operation principle (i.e., ALEX or FWM) as schematically shown in Fig. 2.

Figure 2 depicts the characteristics of the three CWLDs triggered using the ALEX approach with a trigger-delay among the diodes of *Δt* = 200 ns. The optical pulses shown in Fig. 2a–c were recorded with an averaging of 10^4^ using an ultra-fast metal–semiconductor-metal photodetector. The trigger signal of 16 ns provided by the arbitrary function generators led to optical pulses of 7.89 ns (Fig. 2a; blue; R^2^ = 0.97), 9.43 ns (Fig. 2b; green; R^2^ = 0.92), and 9.50 ns (Fig. 2c; red; R^2^ = 0.94). Furthermore, no after-pulse caused by electric coupling or cross-triggering was observed. The pulse energies as shown in Fig. 2d–f further fluctuated with a standard deviation of 2.3% (Fig. 2d; blue), 3.1% (Fig. 2e; green), and 3.2% (Fig. 2f; red). For all CWLDs, the pulse energies slightly increased over the first ~ 100 pulses, which might be due to reaching a steady-state operation temperature. The achieved pulse energies at the sample position were characterized to be 26 nJ/pulse, 18 nJ/pulse, and 11 nJ/pulse at 100 kHz and 300 V for the blue, green, and red CWLD, respectively. The spatial filtering required for near-diffraction-limited focusing and the subsequent sequence of optics for beam shaping, spectral merging, and guidance through the galvanometric scanning unit and objective lens required an operational power of 189 nJ/pulse, 120 nJ/pulse, and 142 nJ/pulse for the blue, green, and red CWLD, respectively. The actual emitted wavelength depicted in Fig. 2g–i of the three CWLDs was determined to be 440.9 nm at a line width of 1.87 nm (Fig. 2g; blue; R^2^ = 0.99), 513.5 nm at a line width of 4.00 nm (Fig. 2h; green; R^2^ = 0.98), and 635.6 nm at a line width of 1.92 nm (Fig. 2i; red; R^2^ = 0.98), respectively. Analogous characterization using the FWM approach was in very good agreement and exhibited deviations below ~ 5% with respect to the above-mentioned values (see^16,32^ for comprehensive characterizations).

**Figure 3:**
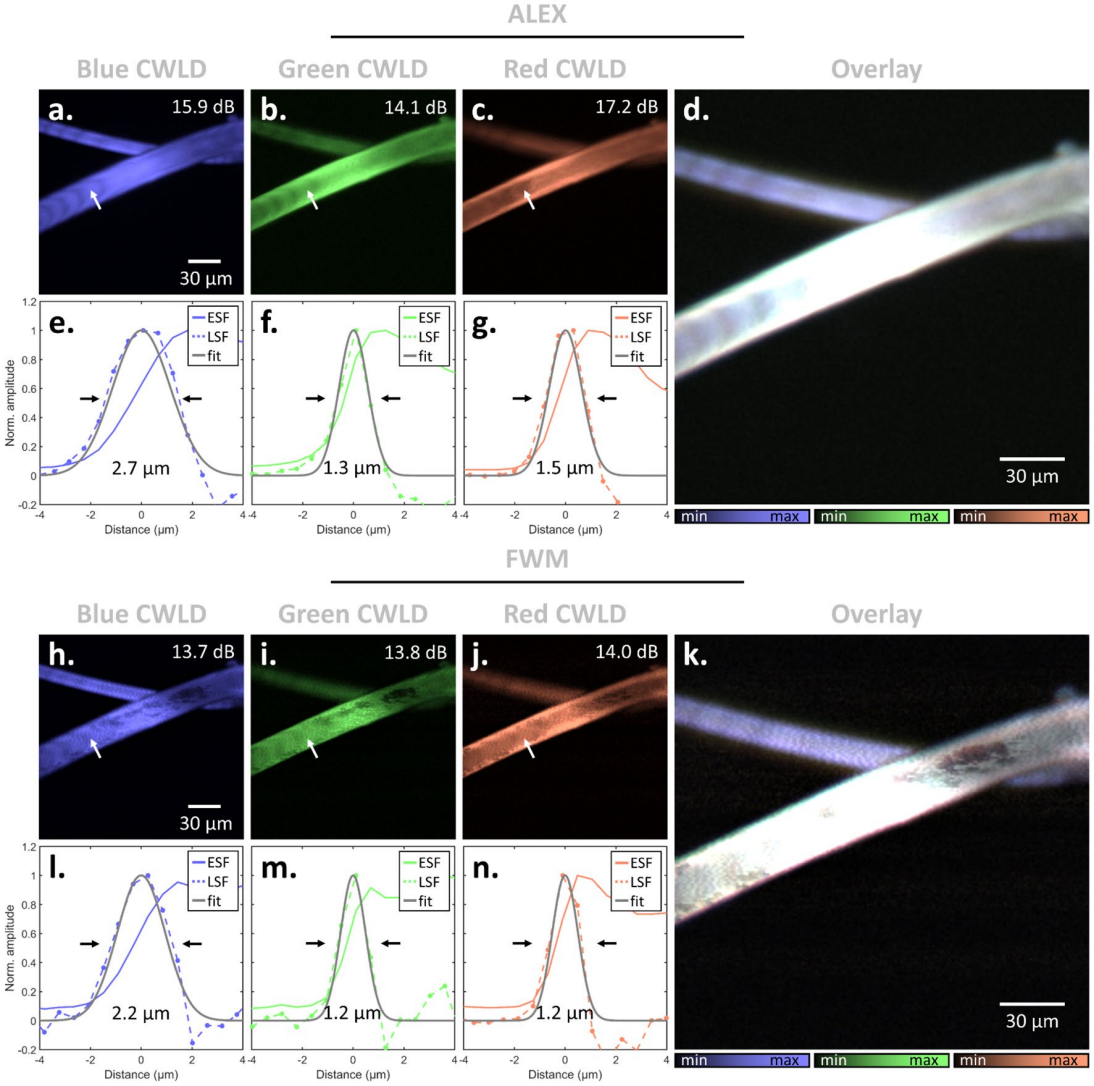
Imaging performance of ALEX and FWM optoacoustic microscopy in phantoms. Two intertwined sutures of 18 μm and 30 μm diameter were used. (**a**–**c**) Separate depiction of simultaneously recorded images in ALEX mode of the (**a**) blue, (**b**) green, and (**c**) red CWLD, and their (**d**) overlay. (**e**–**g**) Achieved resolutions determined by extracting the respective edge-spread-functions (ESF) along the arrows indicated in (**a**–**c**) and Gaussian fitting the corresponding line-spread-functions (LSF). (**h**–**n**) Analogous depictions and analysis of the FWM measurement of the identical sample.

Next, we tested the imaging capabilities using a reference phantom of two intertwined black sutures of 18 μm and 30 μm diameter, shown in Fig. 3. After beam collimation and co-alignment of the laser foci, the sutures were imaged with a 10 × 0.45 NA objective using the ALEX approach (see Fig. 3e–g) and the FWM approach (see Fig. 3h–n), both carried out with all three CWLDs operating simultaneously and their signals separated as described previously. In both operation modes, the sutures were homogeneously revealed by all three CWLDs. The achieved image SNRs (defined as $SNR = \mu_{Sig} /\sigma_{bg}$ with $\mu_{Sig}$ denoting the average signal intensities after image reconstruction and ${\sigma }_{bg}$ the standard deviation of the background) were 15.9 dB (Fig. 3a), 14.1 dB (Fig. 3b), and 17.2 dB (Fig. 3c) for ALEX, and 13.7 dB (Fig. 3h), 13.8 dB (Fig. 3i), and 14.0 dB (Fig. 3j) for FWM, for the blue, green, and red CWLD, respectively. The lower SNR yielded for FWM might be due to capturing electric noise in generating optoacoustic signals at excitation rates of > 1 MHz. The overlays for ALEX (Fig. 3d) and FWM (Fig. 3k) measurements revealed good spatial coalignment of the CWLD beams. The yielded resolution, as plotted in Fig. 3e–g and Fig. 3l–n, was determined via Gaussian fitting of the line-spread-function (LSF), which constitutes the first derivative of the edge-spread-function (ESF) over an arbitrary sharp boundary, thereby revealing the achieved spatial resolution. The obtained resolutions were 2.68 μm (Fig. 3e; blue; R^2^ = 0.95), 1.34 μm (Fig. 3f; green; R^2^ = 0.89), and 1.50 μm (Fig. 3g; red; R^2^ = 0.82) for ALEX, and 2.22 μm (Fig. 3l; blue; R^2^ = 0.93), 1.16 μm (Fig. 3m; green; R^2^ = 0.89), and 1.19 μm (Fig. 3n; red; R^2^ = 0.88) for FWM, respectively. The higher resolution achieved in FWM may be due to the 100-fold lower scanning speed of the galvo mirrors and, thus, a higher precision of the optical foci positioning.

Finally, we investigated the in vivo applications of simultaneous multi-wavelength optoacoustic microscopy by animal imaging. To showcase the capabilities and flexibility of the CWLD-based optoacoustic microscope, we carried out experiments in both operational modes, i.e., ALEX and FWM, each capitalizing on the simultaneous multi-wavelength imaging. In particular, we used ALEX-imaging to monitor injected melanocytes, and FWM-imaging to monitor oxygenation dynamics during an oxygen stress challenge in mouse ears. This imaging target was selected as it is a typical tissue where optoacoustic implementations are conventionally demonstrated on.

In the first experiment, three mice were administered ~ 10^6^ B16F10 murine metastatic melanoma cells via tail vein injection and subsequently imaged. Whereas the absorption spectrum of hemoglobin coincided with the wavelengths of the blue and the green CWLDs, it exhibited a negligible absorption for the red CWLD. Hence, the red CWLD was dedicated for revealing B16F10 cells circulating in the vascular system.

**Figure 4:**
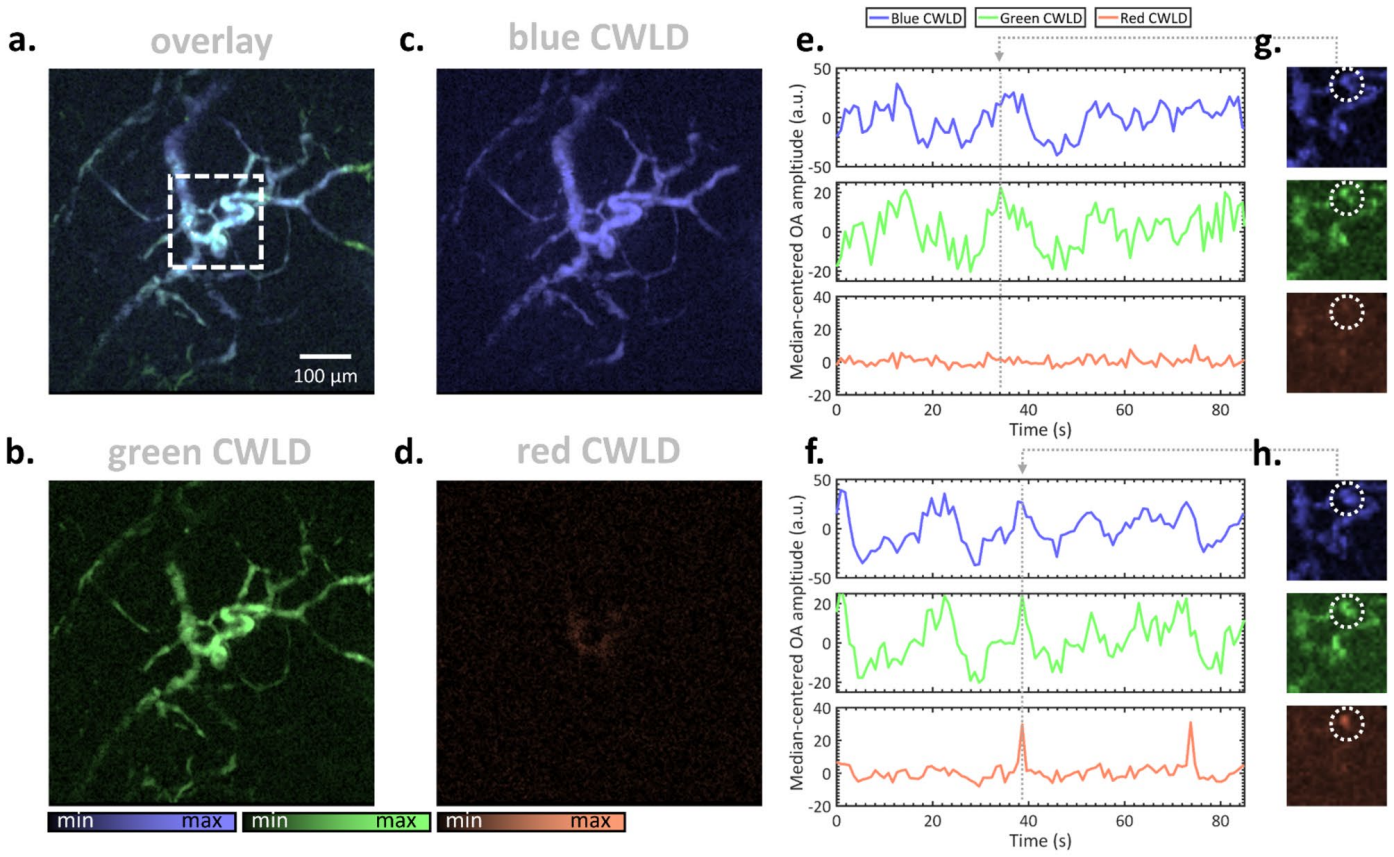
Multi-wavelength microscopy of circulating melanocytes in vivo. Mouse ears were imaged using overdriven CWLD in ALEX operation mode. (**a**–**d**) High-resolution images depicted (**a**) overlaid or (**b**–**d**) separately for the respective CWLDs. (**e**) OA signal trajectories averaged across the circle as indicated in the selected (**g**) frames of the ROI marked in (**a**). (**f**–**g**) Analogous plots and depiction after tail vein injection of B16F10 melanoma cells.

Figure 4 shows ALEX-imaging of the mouse ear before and after injection of B16F10 melanocytes to mimic circulating tumor cells. Figure 4a depicts the overlaid images acquired with the three CWLDs before melanocyte injection, which are separately shown in Fig. 4c, d. Here, the blue and green CWLDs revealed the microvasculature of the mouse ear in reasonable detail, whereas the red CWLD hardly contained any signal in the center of the field of view (FOV). Figure 4e plots OA signal trajectories of the vessel marked with a circle in the selected high-speed frames in Fig. 4g. The trajectories recorded with the blue and green CWLDs as well as their selected high-speed imaging frames resembled each other, indicating similar contrast originating from hemoglobin in circulating red blood cells (RBC). The trajectory and selected frame of the red CWLD solely contained the background signal. Figure 4f–h analogously illustrate the data acquired after tail vein injection of melanocytes. Whereas the blue and green CWLDs again displayed similar time-wise trajectories and spatial signal patterns, the red CWLD contained two peaks in the trajectory, which probably originated from a single B16F10 cell passing through the interrogation area as shown in the associated frame. These proof-of-concept imaging attempts suggest successful discrimination of RBCs and circulating B16F10 melanocytes via simultaneous multi-wavelength optoacoustic microscopy.

In the second experiment, we subjected an anesthetized mouse to oxygen-stress conditions by changing the carrier gas of the anesthesia from 100% O_2_ to air (i.e., 20% O_2_) every 30 s. Since the isosbestic point of the hemoglobin absorption spectra lies between the emission wavelengths of the blue and green CWLDs, we opted for monitoring the oxygenation of hemoglobin using the ratio of their signals.

**Figure 5:**
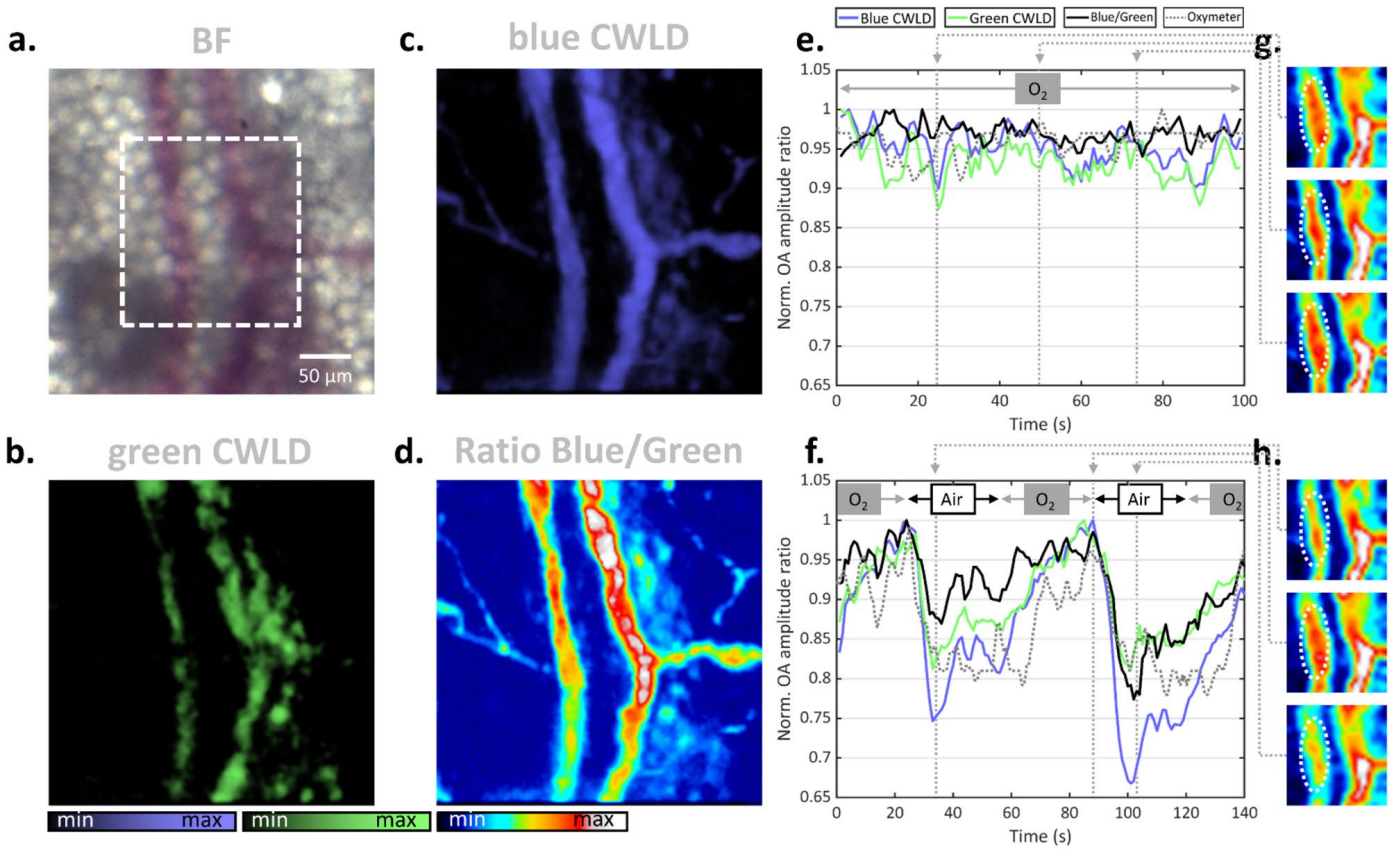
Multi-wavelength microscopy of oxygenation dynamics in vivo. (**a**–**d**) Mouse ears after an oxygen stress test were imaged using overdriven CWLD in FWM operation mode. High-resolution images depicting (**a**) brightfield, (**b**, **c**) the respective CWLDs, and (**d**) normalized ratio. (**e**) OA signal trajectories and simultaneously recorded oximeter values averaged across the ellipse as indicated in the selected (**g**) frames of the ROI marked in (**a**). (**f**, **g**) Analogous plots and depiction during 30 s oxygenation stress tests.

Figure 5 depicts FWM imaging of a mouse ear during standard anesthesia as well as during oxygenation stress challenges. Figure 5a shows a brightfield image across the FOV subsequently imaged with the CWLD in FWM mode as shown in Fig. 5b and c, the ratio of which is shown in Fig. 5d. In here, two vessels can be identified as a vein and artery. Figure 5e plots OA signal trajectories of the vessel marked with an ellipse in the selected high-speed frames in Fig. 5g acquired within the ROI marked in Fig. 5a. The trajectories of the blue and green CWLD resembled each other indicating the sensing of the same moiety. Furthermore, both their ratio as well as the simultaneously recorded blood oxygenation using a pulse-oximeter remained unchanged over the recorded time window. Figure 5f–h analogously depict data during an oxygenation stress challenge in which the carrier gas for the anesthesia was alternated between 100% O_2_ and 20% O_2_ every 30 s. The two OA signal trajectories, their ratio, as well as the oximeter curve resemble each other following a saw tooth function. The data highlight the capability of monitoring blood oxygenation using simultaneous multi-wavelength optoacoustic microscopy.

## Discussion

We demonstrate a novel concept for optical-resolution optoacoustic microscopy that utilizes three overdriven continuous-wave laser diodes as novel excitation sources for multi-wavelength imaging. Our results outline how low-cost and miniaturized laser diodes can offer similar or superior performance in OR-OAM regarding speed, signal-to-noise ratio, and resolution compared to the conventionally used bulky and costly lasers and pave the way for affordable and portable systems.

We developed an optical-resolution optoacoustic system that allows, for the first time, truly simultaneous multi-wavelength imaging on the microscale based on semiconductor light sources. We capitalized on the small light-emitting area of three CWLDs, thereby providing a near-diffraction-limited focusable beam profile, and yielded high spatial resolution below ~ 2 µm, which matches the best-achieved performance of optoacoustic microscopy with diodes. By precisely adapting the optical components for each equipped CWLD, we have achieved, for the first time, multi-wavelength OR-OAM microscopy using three excitation wavelengths co-aligned with micrometer precision, whereas previous studies have only used one wavelength.

We further capitalized on the custom-designed adaptable-triggering engine (ATE), which enabled precise temporal synchronization of the three CWLDs allowing truly simultaneous multi-wavelength OR-OAM. Based on repetition rates of up to ~ 1 MHz, which is 33-fold higher^17^ than the current state-of-the-art, we attained frame rates beyond 5 Hz for multi-wavelength imaging across standard microscope fields of view. In addition, the ATE facilitated the application of highly flexible triggering schemes that permit, for the first time, the ability to characterize and compare different acquisition modes using identical system components. We tested alternating laser excitation and frequency-wavelength multiplexed and found their performance practically identical in resolution, SNR, and speed. Such information is not accessible by other OR-OAM microscopes, which typically only allow one acquisition mode due to their slow wavelength-tuning or fixed and low repetition rate.

We employed the novel imaging capabilities of multi-wavelength optoacoustic microscopy at high spatial and temporal precision to monitor and discriminate blood cells and melanocytes in vivo, with the latter mimicking circulating tumor cells. Further, we capitalized on the equipped diodes’ emission wavelengths to monitor blood oxygenation in response to an oxygenation stress challenge. These results demonstrate the capability of our system to monitor dynamic biological processes with similar, or even higher, fidelity when compared to traditional laser sources, while offering significant advantages in cost, footprint, and versatility.

This work demonstrates the potential of overdriven laser diodes to develop cost-effective and versatile multi-wavelength optical-resolution optoacoustic systems for biological and biomedical applications. Due to the low cost, small size, and broad wavelength ability of the continuous-wave laser diodes, and the use of a custom-build control unit, the future integration of more wavelengths for interrogating additional intrinsic contrast in tissue promises to be straightforward. Further, the flexible acquisition mode combined with the miniature footprint facilitates the development and benchmarking of new multiplexing schemes, especially when targeting specific research questions or prototyping handheld devices. Despite meeting the requirements of providing stable optical excitation, CWLDs are not available at every wavelength, due to the need for the development of new materials to enable new wavelengths. This limitation can make it harder to target the maximum absorption peak of a specific chromophore. Another limitation of CWLDs is the relatively low pulse energy and multi-mode output, complicating the optical assembly for achieving efficient diffraction-limited focusing. However, taking the accelerating improvements of laser diodes into account, we expect new wavelength, single-mode and high-power output laser diodes to become available in the near future.

In summary, laser diodes constitute a highly promising alternative excitation source for optoacoustic microscopy by reducing the cost and size of the excitation source while providing high SNRs, frame rates, and multi-wavelength imaging for biological applications. The vast potential to adapt the optical assembly and acquisition procedure of diodes for optoacoustic sensing facilitates the construction of portable and small footprint devices, while streamlining the customization of their capabilities to a given imaging problem—broadening the scope of optoacoustic applications on the microscale.

## Methods

### Electrical and optical setup

All CWLDs were driven by a custom-designed electrical circuit (see inset in Fig. 1a) as comprehensively described in^16^. In short, the capacitor C was first charged at ~ 300 V connected to the RC–C–D1 circuit, and then discharged over the laser diode upon receiving an ultrashort trigger signal on the MOSFET Q1. RCL served here to limit the current, which was controlled through the applied high voltage. The rise and fall time of the achieved current pulse was determined by the turn-on speed of MOSFET Q1 and the time constant of the RCL–C–LD–Q1, respectively. This driver finally achieved current pulses shorter than ~ 10 ns of up to ~ 50–60 A at repetition rates surpassing ~ 500 kHz. Such drivers ultimately controlled a CWLD module each (PD-01236, 6W, 445 nm; PD-01289, 1W, 520 nm; PD-01229, 700 mW, 638 nm, Lasertack, Germany). All three diodes were charged by a high-voltage generator to ~ 300 V (EA-3050B, EA, Germany) and cooled by a heat sink combined with an attached fan. The temperature was monitored by a custom-programmed Arduino platform and always kept below 60 °C.

The CWLDs were integrated in an existing optical-resolution optoacoustic microscope as described comprehensively in^24–31,33^. All CWLDs were held by a kinematic platform (KM100B, Thorlabs) for beam alignment. The emitted beam of each diode was guided through a telescopic arrangement of plano-convex lenses equipped with a 50 μm high-power pinhole for collimation, beam cleaning, and size adjustment. The merging of the beams was achieved by longpass dichroic mirrors (DMLP490R and DMLP550R, Thorlabs) before encountering a set of high-precision galvanometric (galvo) mirrors (6215H Galvanometer Scanners, Cambridge Technologies) in combination with an inverted microscope stand (AxioObserver.D1, Zeiss) for laser-scanning. The optoacoustic signals were acquired using a 50 MHz transducer (HFM23, Sonaxis) controlled by a custom-developed triggering method (see “Triggering and scanning”) in a streaming-like acquisition mode (see^24,27,30,33^ and “Data recording”).

### Triggering and scanning

The trigger-TTL-signals for the CWLDs were provided by two electrically synchronized high-speed arbitrary waveform function generators (AWG) (33622A, Keysight Technologies, USA), which themselves receive the trigger signal provided by a data acquisition card connected to a BNC connector block (BNC-2110, National Instruments; 16 analogue input/output connections, 2 digital input/output connections, range: ± 10 V), which also send out two analogue waveforms ranging across ± 2 V as the slow-axis and fast-axis scanning curves for the galvo mirrors. Using two AWGs being triggered by the identical trigger signal, which is also used for data acquisition but operates here independently, served as an adaptable-triggering engine (ATE) that allowed us to carry out multi-wavelength optoacoustic microscopy based on two approaches, namely ALEX and FWM, as schematically illustrated in Fig. 1b. ALEX led to firing all diodes sequentially one after another upon each global trigger signal. The delay between the three timepoints of the actual laser pulses was set to *Δt* = 200 ns. The frequency-power-spectrum of the optical excitation contained the underlying global trigger signal repetition rate *f*_*rep*_ as well as higher harmonic occurrences. Due to *f*_*rep*_ ≈ 100–200 kHz, the data were acquired exclusively at *f*_*rep*_ and contained time-shifted OA signals corresponding to the excitation of the three CWLDs. Based on the applied *Δt *in their time-point of emission, the OA signals could then be separated from each other by time-windowing. FWM drove the CWLDs in bursts of 100 trigger signals upon receiving a trigger signal (i.e., 1 kHz) at a burst repetition rate of *f*_*b*_ *+ n·Δf*, in which *f*_*b*_ was set to 1 MHz, Δf to 4 kHz, and n was 0, 1, or 2 for the respective diodes. As such, every 1 ms the CWLDs sent out 100 pulses at 1000 kHz for the 445 nm diode, at 1004 kHz for the 520 nm diode, and at 1008 kHz for the 638 nm diode. Hence, the frequency-power-spectrum contained three sets of frequency harmonics, in which their respective peak locations deviate at increasing frequency harmonics. Upon each global trigger signal, a time-window was acquired that captured the entire bursts of OA signals, which were then separated in the frequency domain based on their distinct burst repetition rate. For appropriate comparison between ALEX and FWM, the measurement time and averaging were set equally. Whereas ALEX measurements were performed at a global trigger signal repetition rate of 100 kHz and an averaging of 500, FWM measurements used a global trigger signal repetition rate of 1 kHz, a burst averaging of 5, and 100 pulses per burst. This led for both approaches to a pixel dwell time of 5 ms with 500 pulses being used for imaging.

### Data recording

Synchronization and data acquisition were facilitated by the use of two data acquisition cards, hereinafter called DAQ1 and DAQ2 (DAQ1: PCIe 6363, National Instruments; max. sampling rate: 1 MS/s; and DAQ2: ADQ412, SP Devices; max. sampling rate: 3.6 GS/s). The entire scanning procedure was programmed in a custom-developed MATLAB™ script. DAQ1, which was connected to a BNC connector block (BNC-2110, National Instruments; 16 analogue input/output connections, 2 digital input/output connections, range: ± 10 V), which sends out a trigger signal that determines the scanning frequency, the acquisition frequency, as well as the repetition rate of actively triggered lasers for optoacoustic microscopy and constitutes a 0–5 V TTL signal as a digital waveform. DAQ1 further sent out two analogue waveforms ranging across ± 2 V as the slow-axis and fast-axis scanning curves for the galvo mirrors. DAQ2 was used for reading the high-speed optoacoustic signals. For synchronization purposes, the trigger signal provided by DAQ1 was also fed into the trigger input of DAQ2. As DAQ2 ran at a very high sampling frequency and accepted incoming signals in the range of ± 750 mV, electrical attenuators and a high-pass filter (EF515, Thorlabs) were installed to shape the incoming TTL signal of 50% duty cycle appropriately to a sharp trigger peak. Hence, both data acquisition cards were electrically synchronized. Based on ~ 200 MHz being the highest frequency expected to be sensed by the equipped ultrasound transducers, the sampling rate of DAQ2 was set to 450 MS/s in order to fulfil the Nyquist–Shannon sampling theorem. Further, a streaming-like acquisition mode was developed being only restricted by the data transfer rate of the PCIe-connection of DAQ2 to the computer (i.e., 3.2 GB/s) and the memory capacity of the computer (i.e., 64 GB in case of the used PC). The streaming-like acquisition mode was based on assigning temporary data buffers internally on the DAQ2 memory, in which the recorded data can be continuously filled (see^24,27^).

### Signal separation

The excitation source was modulated in time using a train of pulses at a specific base repetition rate. This means that, in the frequency domain, we excite frequencies that are all integer multiples of the base repetition rate, i.e., its harmonics. The optoacoustic signal was recorded in the time domain and then transformed into the frequency domain using the Fourier Transform for each frequency that was excited, i.e., the harmonics of the base repetition rate for the blue, green, and red laser diode, respectively. This means that we discard every other frequency, that contains only noise, other than the ones into which we know the signal is concentrated. Then using only three frequencies, we perform the inverse Fourier transform to retrieve the OA signal, now averaged as proven here in the time domain.

### Animal model

All animal procedures were approved by the Government of Upper Bavaria; Veterinary Office, Maximilianstrasse 39, 80538 Munich, Germany. All animal experiments were conducted in accordance with all relevant guidelines and regulations, and with ARRIVE guidelines. The in vivo experiments were performed using athymic nude Hsd Foxn1 mice. An automated physiological monitoring system was installed for mouse in vivo measurements (PhysioSuite, Kent Scientific). This system monitored heart- and breathing-rate, blood oxygenation, and body temperature of the mice. The latter was controlled in a fully automated feedback scheme using heating foils (thermo polyester heating foils, Conrad) being placed beneath the mice. Furthermore, a small animal anesthesia unit (Tabletop Veterinary Anesthesia Machine, Keebovet) was used for mouse in vivo measurements. During measurements, the mice were anesthetized with ~ 2% isoflurane and placed onto a dedicated mouse holder. The end pieces of both the monitoring and the anaesthesia system for mouse in vivo measurements were fixed to a custom-designed mouse holder fitted on the sample holding stages. This holder contained an elevated pedestal comprising a central hole across which a 170 μm cover slip was placed. On top of the cover slip, the mouse ear was placed and fixed. A drop of ultrasound gel could be pressed on top of the ear with a thin polyethylene foil wrapped around a hollow frame, which was screwed to the aforementioned pedestal. In this condition, the mouse ear was placed between the glass cover slip from below and the ultrasound-gel-pressing foil from above. This allowed the ear to be accessed optically from below by elevating the equipped microscopic objective lens and accessed acoustically from above by lowering the ultrasound transducer into the hollow frame filled with water. The holder was iteratively optimized using a 3D printer (RF1000, Renkforce) and finally fabricated from aluminum to enhance stability.

## Data Availability

Data underlying the results presented in this paper are not publicly available at this time but may be obtained from the corresponding author upon reasonable request.
